# Cadence-Based Pedometer App With Financial Incentives to Enhance Moderate-to-Vigorous Physical Activity: Development and Single-Arm Feasibility Study

**DOI:** 10.2196/56376

**Published:** 2024-10-24

**Authors:** Kosuke Hayashi, Hiromitsu Imai, Ichiro Oikawa, Yugo Ishihara, Hirokazu Wakuda, Iori Miura, Shingo Uenohara, Asuka Kuwae, Megumi Kai, Ken'ichi Furuya, Naoto Uemura

**Affiliations:** 1 Department of Clinical Pharmacology and Therapeutics Oita University Yufu Japan; 2 Department of Medical Ethics Faculty of Medicine Oita University Yufu Japan; 3 Clinical Pharmacology Center Oita University Hospital Yufu Japan; 4 Faculty of Science and Technology Oita University Oita Japan

**Keywords:** physical activity, behavioral economics, pedometer, arm, cadence, app, public health, walk, Google Fit, heart points, exercise, mobile phone

## Abstract

**Background:**

High levels of physical activity are key to improving health outcomes, yet many people fail to take action. Using pedometers to target steps per day and providing financial incentives is a simple and scalable approach to promoting public health. However, conventional pedometers do not account for “intensity” and “duration,” making it challenging to efficiently increase people’s moderate-to-vigorous physical activity (MVPA), which is expected to improve health outcomes. Based on these rationales, we developed a smartphone app that sets step cadence as a goal (defined as a daily challenge of walking more than 1500 steps in 15 minutes twice a day, which is a heuristic threshold for moderate physical activity) and provides financial incentive when the challenge is met.

**Objective:**

This study aimed to evaluate the feasibility of our novel app and explore whether its use can increase users’ daily MVPA.

**Methods:**

A single-arm pre-post study evaluated the feasibility and efficacy of the app. A total of 15 participants used app 1 (an app without financial incentives) for the first period (4 weeks) and then switched to app 2 (an app with financial incentives) for the second period (4 weeks). The primary outcome was the difference between the first and second periods in the number of successful challenge attempts per week. Secondary outcomes were differences between the first and second periods in daily steps and distance walked. Exploratory outcomes included the difference between the first and second periods in daily “heart points” as measured by Google Fit, a publicly available app that measures users’ daily MVPA.

**Results:**

The number of successful challenge attempts per week increased significantly compared to the first period (5.6 times per week vs 0.7 times per week; *P*<.001). Although not statistically significant, there was a trend toward an increase in the mean steps per day and distance walked per day (6586 steps per day vs 5950 steps per day; *P*=.19; and 4.69 km per day vs 3.85 km per day; *P*=.09, respectively). An exploratory end point examining daily MVPA by “heart points” collected from Google Fit also showed a significant increase compared to the first period (22.7 points per day vs 12.8 points per day; *P*=.02).

**Conclusions:**

Our app using step cadence as a goal and providing financial incentives seemed feasible and could be an effective app to increase users’ daily MVPA. Based on the results of this study, we are motivated to conduct a confirmatory study with a broader and larger number of participants.

**Trial Registration:**

UMIN 000050518; https://center6.umin.ac.jp/cgi-open-bin/ctr/ctr_view.cgi?recptno=R000057420

## Introduction

High levels of physical activity are key to improving and preventing lifestyle-related diseases, yet many people fail to take action. Various guidelines for lifestyle-related diseases, such as hypertension and diabetes mellitus, recommend at least 30 minutes per day or 150 minutes per week of moderate-intensity exercise [1,2]. Since these diseases are responsible for numerous deaths and skyrocketing health care costs, “exercise” that is safe and inexpensive should be prescribed as a medicine to almost the entire population. However, the prevalence of physical inactivity in developed countries was reported to be as high as 36.8% in 2016 [3]. With the World Health Organization’s target of a 10% relative reduction in the prevalence of physical inactivity for the prevention of noncommunicable diseases [4], this unmet need must be addressed.

Digital therapeutics are gaining attention as a solution to address this issue. For instance, apps designed to educate patients on lifestyle modifications have proven effective in reducing blood pressure [5] and hemoglobin A_1c_ [6] in clinical trials and are approved for clinical use, although challenges with long-term app adherence persist in the real world [7,8].

We believe that a potential reason why lifestyle modifications are underused by the public could be explained by the so-called present bias, in which people prioritize immediate pleasure (such as sedentary behavior) over long-term benefits (such as better health and longevity) when the benefits of having healthy behavior are in the distant future [9]. In recent years, behavioral economic methods have been introduced in the medical field to overcome present bias and promote behavioral change. For instance, interventions that use financial incentives to motivate people to act, such as smoking cessation [10] and weight loss [11], are gaining increasing attention. Several reports have also shown that financial incentives are effective in increasing the number of steps taken per day [12,13]. Furthermore, several studies have investigated the effectiveness of different financial structures, such as gain frame incentives, loss frame incentives, and lottery incentives [14]. Loss frame incentives are based on the theory of loss aversion, designed with the understanding that people feel the pain of losing something more than the joy of gaining it. Lottery incentives, on the other hand, are designed to exploit the psychological tendency to overestimate small probabilities. As behavioral economics gains attention in the medical field, these approaches may also provide a solution for improving long-term adherence to digital therapeutic apps, which also face issues related to present bias.

When financial incentives are integrated into the app, clear criteria for reward must be established. Using pedometers to track steps and provide financial incentives offers a simple and scalable method for the public to enhance physical activity. However, focusing solely on step count overlooks factors such as intensity and duration of walking. Moderate physical activity (MPA) is particularly important for improving health outcomes, yet relying solely on step counts makes it challenging to effectively increase MPA [15]. Wearable devices are gaining attention since they can measure daily moderate-to-vigorous physical activity (MVPA) [16], but their cost and the necessity of continuous wear limit their scalability to the broader public.

In comparison to wearable devices, standalone smartphone apps present a scalable solution due to the increasing penetration rate of smartphones [17]. From these rationales, we developed a pedometer app that simply measures walking intensity by step cadence and time and provides financial incentives based on these measurements (we defined achievement of MPA based on cadence as at least 100 steps per minute). The concept of the novel app was to be a tool to increase MVPA more efficiently than conventional pedometers and to be more scalable to the masses. Our ultimate goal is to investigate the therapeutic effects of the app on lifestyle-related diseases. However, we considered the present trial as equivalent to phase 1 studies in drug development and thus decided to start the trial with relatively healthy participants. The primary objective of this study was to evaluate the feasibility of the concept of our novel app and to explore whether its use can increase users’ daily MVPA. Our primary hypothesis was that our novel pedometer app targeting step cadence as a goal with financial incentives is feasible in a way that the users will at least use the app and it could be used for further clinical trials. Our secondary hypothesis was that app use can lead to an increase in people’s daily physical activity.

## Methods

### Features of the Smartphone App

#### Overview

**Figure 1:**
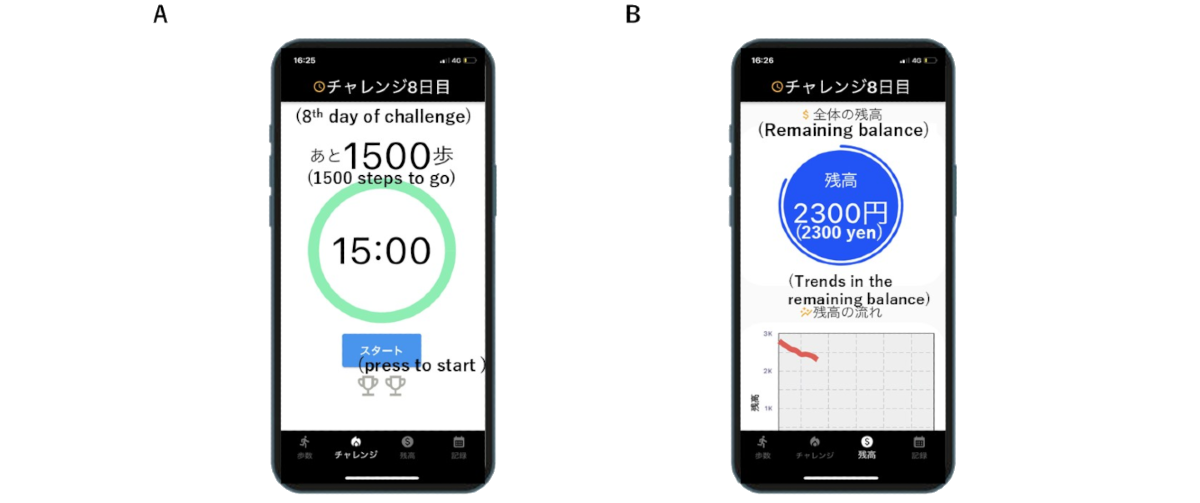
The developed app’s interface is displayed. Panel (A) represents the daily challenge function. Panel (B) represents the function that enables users to track their current incentives earned during the study. The number in the green circle shows the time remaining for the challenge (15 minutes in this image). The start button, located at the lower part of the screen, can be pressed to begin. The number above the green circle indicates the remaining steps needed to accomplish the challenge. The number in the blue circle in panel (B) represents the maximum potential earnings during the remaining study period (JP¥ 2300 in this image; US $20.16). The chart in the lower half of the screen illustrates the trend of decreasing balances.

The smartphone app was developed by the study team at the Faculty of Science and Technology, Oita University, based on specifications prepared by the study team at the Faculty of Medicine, Oita University. The concept was to form a team capable of conducting early exploratory app development in the medical field led by a board-certified internal medicine physician. Study team members from the Department of Medicine included internal medicine physicians, clinical pharmacology physicians, and pharmacologists specializing in early phases of drug development, anticipating that their expertise in early drug development could be applied to early medical app development. Team members from the Faculty of Science and Technology had research experience in medical device and smartphone app development. The mobile app was coded using Flutter (version 3.7.10; Google) and consists of 2 main components (Figure 1).

#### Daily Challenge Function

**Figure 2:**
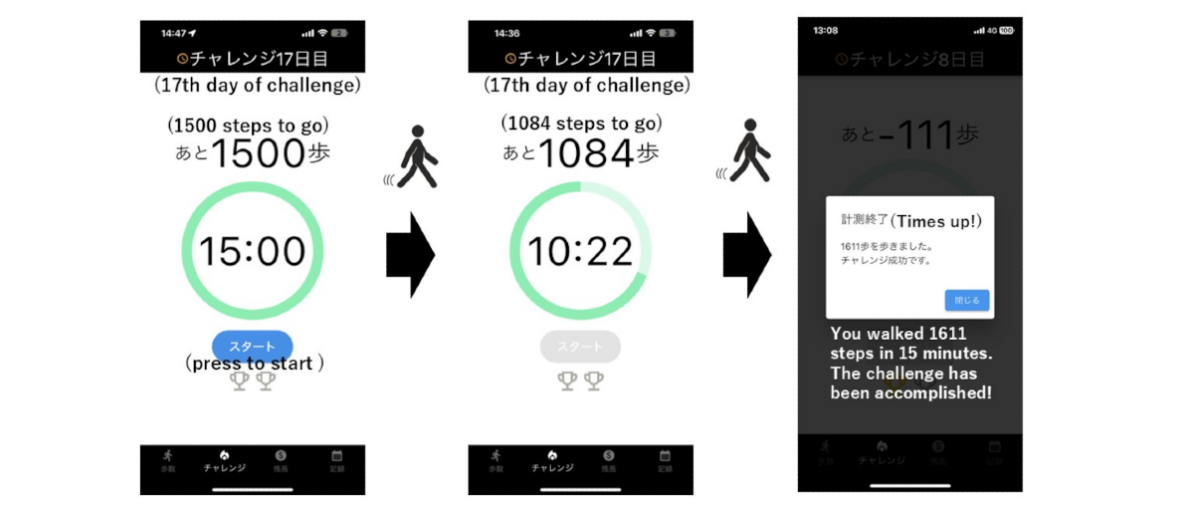
The user journey of the app’s challenge function is depicted. Upon pressing the “start” button located at the lower part of the screen, the timer inside the green circle begins a countdown (initially set to 15 minutes). The numbers above the green circle indicate the remaining steps required to complete the challenge before the timer expires (initially set to 1500 steps). An example illustrating the progress of the challenge is presented in the middle of the figure (in this case, indicating the need to walk 1084 steps within 10 minutes and 22 seconds). Upon successful completion of the challenge, the app announces its accomplishment.

The first component is the “daily challenge” function, where users are encouraged to achieve a specific goal (Figure 1A). We set our “challenge” as walking at least 1500 steps in 15 minutes. This challenge was chosen as the minimum requirement for MPA since several literature reports have already examined the validity of a step cadence of 100 steps per minute or greater as a heuristic for achieving MPA (defined as 3 metabolic equivalents or greater) across various age groups [18]. When the “start” button located in the lower part of the screen is pressed, the timer inside the green circle initiates a countdown (for this study, 15 minutes). The numbers above the green circle indicate the remaining steps needed to complete the challenge before the timer reaches zero (for this study, 1500 steps; Figure 2). Once the challenge is successfully accomplished, one of the 2 cups in the lower half of the screen lights up. The user can complete up to 2 challenges per day, corresponding to 30 minutes of MPA, which is recommended by many guidelines. Users can attempt the challenge as many times as possible until it is achieved. Each time the challenge is met, a financial incentive of JP¥ 50 (approximately US $0.36) is generated, with a maximum of JP¥ 100 (US $0.72) per day.

#### Incentive Tracking Function

The second component allows users to check their current incentives earned during the study (Figure 1B). We used a loss-framed financial incentive design for this component. At the start of app use, JP¥ 2800 (US $20.16, which is the theoretical maximum incentive that could be earned in 4 weeks of participation with an assumption that users are agreeing to 2 walking challenges per day) was virtually deposited into the app and displayed on the screen. If the daily goal is met, the amount remains unchanged; if not, the potential incentive for that day is deducted from the displayed amount. We adopted this design since previous research has shown that people are more motivated by losses than gains [19].

Two types of apps were developed for the study to evaluate the effect of the loss-framed financial incentive concept: (1) “app 1,” which included the challenge function but lacked financial incentives, and (2) “app 2,” which included all features including the challenge function and financial incentives.

### Study Design Overview

This was an 8-week single-arm, open-label study. All participants who enrolled in the study used app 1 for the initial 4 weeks and then switched to app 2 for the subsequent 4 weeks. End points were measured every 4 weeks after using each app and pre-post differences were examined. We opted for a sequential (pre-post) comparison study instead of a crossover study due to concerns about potential carryover effects and uncertainty regarding the optimal washout period.

### Ethical Considerations

The study complied with the Declaration of Helsinki and the Japanese Ethical Guidelines for Clinical Research and was approved by the Clinical Trial Ethics Committee of Oita University Hospital (B22-006). This study was registered at the University Hospital Medical Information Network Clinical Trials Registry (UMIN 000050518) before the inclusion of participants. All participants provided written informed consent prior to study enrollment. Actual incentives earned by the participants during the study period were paid in cash after the trial. The participants’ data collected during the study were deidentified.

### Study Participants

From April 2023 to July 2023, flyers informing about the study were distributed throughout the university. Participants interested in the study contacted the investigator to be determined for eligibility. Eligible participants (1) were aged 18 to 60 years, inclusive; (2) had a BMI of 18 to 30 kg/m^2^, inclusive (which allowed the inclusion of normal weight to overweight participants); (3) owned an iPhone (Apple) and carried it daily; and (4) were able to walk independently without the use of a walking aid.

Participants were excluded if they (1) already exercised at least 3 days per week, (2) were prohibited from exercising by a physician for any reason, (3) had a systolic blood pressure of 160 mm Hg or higher or a diastolic blood pressure of 110 mm Hg or higher at the time of inclusion, or (4) were pregnant or lactating.

### Study Procedure

Participants enrolled were first instructed to download app 1 and also the Google Fit app developed by Google, which is available to the public. The Google Fit app was downloaded since it can measure users’ MVPA based on motion detection built into the phone and record it as “heart points.” For example, 1 minute of brisk walking, defined as 100 steps per minute or more, is recorded as 1 heart point, and 1 minute of running, defined as 130 steps per minute or more, is recorded as 2 heart points [20]. The previous study reported that the ability of Google Fit to count steps was accurate [16]. Participants were encouraged to use the app developed by Oita University daily although they were prohibited from opening the Google Fit app during the study to avoid any bias from its use. Participants were also requested to provide step count and walking or running distance data up to 3 weeks prior to the day of consent was obtained from the Health app (a standard iPhone app) at the time of inclusion. Every day, data, such as total step count, walking or running distance, and the number of challenges achieved, were sent from the app to the university’s server which only the investigators could access. Additionally, data from the Google Fits in-app data; specifically, heart points earned per day, were manually collected during each visit. After 4 weeks of using app 1, the app was deleted and replaced with app 2. While using app 2, the same types of data were sent to the server every day, except for the remaining balance for that day, which was additionally sent to the server. Google Fit was installed at all times during the study period. At the end of the study, all university-developed apps were completely deleted from the participant’s iPhone.

### Outcomes

The primary outcome was the difference between the first (using app 1 without financial incentives) and second (using app 2 with financial incentives) periods in the number of successful “challenge” attempts (walking at least 1500 steps in 15 minutes) in a week. Secondary outcomes were differences between the first and second periods in daily steps and distance walked. Exploratory outcomes included the difference between the first and second periods of daily “heart points” measured by Google Fit. Data for the first week of each period were excluded to avoid an upward bias caused by the initial use of the app. Therefore, the average of the data from weeks 2 to 4 was used for the analysis of each period.

### Statistical Analysis

As this was a proof-of-concept study of an exploratory purpose, no formal sample size calculation was performed. Instead, a sample size that was considered feasible was selected. For usability studies, 80% of the issues can be identified by the first 5 users, whereas 15 participants are sufficient for identifying almost all problems [21]. This was not a mere usability study, although our sample size was selected based on these rationales. All participants enrolled in the study were included in the analysis. Data were expressed as the mean (SD) for continuous variables and numbers and percentages for categorical variables. Hypothesis tests were 2-tailed using a significance level of .05. For the primary end point, the Wilcoxon signed rank test was applied and for the secondary and exploratory end points, a 2-tailed paired *t* test was used to test the difference between pre- and postdata. Analyses were conducted using R (version 4.1.2; R Core Team).

## Results

### Participants’ Characteristics

A total of 15 participants were enrolled between April 2023 and May 2023. All participants were familiar with the operation of common smartphone apps. The mean age of the participants was 22.1 (SD 3.8) years. Approximately half of the participants were female (n=7, 47%). All participants reported a sedentary lifestyle with 0 to 2 physical activity sessions per week. The baseline daily step count (mean step count 3 weeks prior to the study enrollment) was 6268 (SD 2305) (Table 1).

**Table 1:** Baseline characteristics of participants.

Characteristics	Overall (N=15)
Age (in years), mean (SD)	22.1 (3.8)
Sex (female), n (%)	7 (47)
Height (cm), mean (SD)	162.1 (9.8)
Weight (kg), mean (SD)	57.7 (8.0)
Baseline steps per day (steps), mean (SD)	6268 (2305)
Baseline distance walked per day (km), mean (SD)	4.03 (1.41)

### End Points

**Figure 3:**
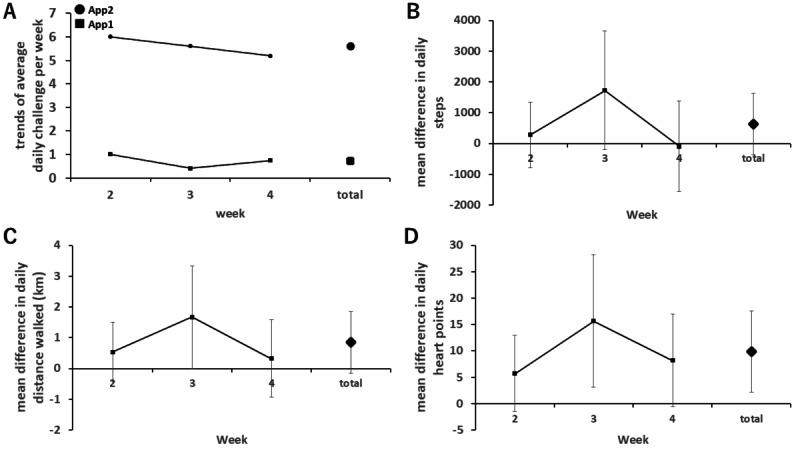
The trends of each end point over 3 weeks are shown as follows; panel (A) illustrates the trends in the average number of challenges accomplished per week between app 1 and app 2. Panels (B), (C), and (D) display the difference in average daily steps, daily distance walked, and daily heart points earned per week between app 1 and app 2, respectively. The error bars represent the 95% CI.

The mean number of successful challenge attempts per week significantly increased compared to the first period (5.6 times per week vs 0.7 times per week; *P*<.001; *r*=30.98; Table 2). Although not statistically significant, there was a trend toward an increase in the mean steps per day and distance walked per day with a medium-sized effect (6586 steps per day vs 5950 steps per day; *P*=.19, *r*=0.34; and 4.69 km per day vs 3.85 km per day; *P*=.09, *r*=0.43, respectively; Table 2). An exploratory end point examining mean daily MVPA time by heart point showed a significant increase (22.7 points per day vs 12.8 points per day; *P*=.02; *r*=0.59; Table 2). Trends at 3 weeks for each end point are summarized in Figure 3. No adverse events were reported during the study.

**Table 2:** Primary, secondary, and exploratory end points^a^.

Variables			App 1		App 2		*P* value
**Primary end point, mean (SD)**							
	Challenge accomplished per week^b^	0.7 (1.3)		5.6 (4.9)		<.001	
**Secondary end point, mean (SD)**							
	Steps per day^c^	5950 (1633)		6586 (1973)		.19	
	Distance walked per day (km)^c^	3.85 (1.16)		4.69 (1.64)		.09	
**Exploratory end point, mean (SD)**							
	Heart points earned per day^c^	12.8 (7.1)		22.7 (12.4)		.02	

^a^All values are represented by the mean of data from weeks 2 to 4. App 1 included the challenge function but lacked financial incentives. App 2 included both the challenge function and financial incentives.

^b^Wilcoxon signed rank test.

^c^Paired *t* test.

## Discussion

### Principal Findings

In this study, we hypothesized that our novel pedometer app targeting step cadence as a goal with financial incentives is feasible and leads to an increase in people’s daily MVPA. To support the hypothesis, following the use of app 2, there was a significant increase in the weekly challenge attempts and daily MVPA as measured by Google Fit’s “heart point” compared to app 1. Although the increases in daily steps and distance walked were not statistically significant, the observed changes suggest that the increase in the weekly number of challenges accomplished directly contributes to users’ daily increase in physical activity since the difference of 5 challenges per week is theoretically equivalent to 7500 steps per week (or 1071 steps per day), which is similar to the observed increase in steps in this study (636 steps). To the best of our knowledge, this is the first study to demonstrate that financial incentives based on step cadence can improve people’s behavior and physical activity. Although efforts to provide financial incentives based on step count have been shown to be feasible and effective in numerous studies [12,13,19], the feasibility of the concept in this study has not been well investigated. The fact that the participants successfully completed the challenge approximately 5 times per week during the second period seems to support the feasibility of our concept. Furthermore, 5 challenge sessions per week are theoretically equivalent to about 10 minutes of MVPA per day. We observed 10 points per day of increase in heart points, which was similar to the expected value. This suggests that performing the challenge directly contributed to the increase in daily MVPA, supporting the validity of the concept. Based on other reports, we believe that this intervention has the potential to reduce the number of deaths per year by 6.9% [22].

The concern with previous reports on providing financial incentives for step counts alone is that the MVPA does not increase sufficiently. For instance, Finkelstein et al [15] reported that providing financial incentives based on weekly step counts was effective in increasing weekly steps compared to a control condition, although the increase in MVPA time was relatively small, averaging around 2 minutes per day compared to baseline. They also failed to improve health outcomes such as blood pressure and body weight.

There are several studies applying loss aversion to incentive mechanisms, with study participants ranging from relatively healthy volunteers to those with obesity to patients with a history of coronary artery disease [13]. However, many of these studies also use step counts as the primary end point, and as far as we are concerned, none have demonstrated effects on true end points such as mortality and disease regression.

The fact that increasing the number of steps alone does not improve health outcomes is not surprising. Masuki et al [23] reported that as the weekly fast walking time increased, estimated peak aerobic capacity (eVO_2_peak) and lifestyle-related disease score improved, but such clinical outcomes were not achieved by slow or total walking time per week. The ultimate goal of an exercise habit is to improve health outcomes, and therefore, a system to provide financial incentives not targeting only steps but the *intensity* and *duration* of walking is necessary. As previously reported, setting a goal of walking at a pace of 3000 steps in 30 minutes per day was effective in increasing daily MVPA compared to a goal of 10,000 steps per day [24]. Although the increase in daily steps in this study was relatively small compared to the conventional study that used the goal of steps per day, the fact that it reached statistical significance in increasing people’s daily heart points may support the effectiveness of the concept of our app.

### Limitations

This study has several limitations. First, the sample size was small and the participants were relatively young. Further studies are needed to test efficacy in a larger number and broader age range of participants. Second, this study’s goal of 1500 steps in 15 minutes may not have provided sufficient intensity for some participants. Setting individualized step cadence goals based on background characteristics may be effective in overcoming this problem. However, because of the simplicity of our current method, we believe it is more scalable to the general population. Third, although this study used Google Fit’s “heart points” to assess MVPA, this is not a validated tool for measuring MVPA. Future research to assess MVPA using a validated tool is desirable. Fourth, while there was a difference in physical activity between app 1 and app 2, other factors, such as self-monitoring and behavioral feedback from the app, may have influenced these results, aside from financial incentives. This warrants further investigation. Fifth, this study was exploratory and had an underpowered design, where *P* values may not fully indicate the robustness of the results. Therefore, further research with appropriate sample size calculations is necessary.

### Conclusions

Our novel app using step cadence as a target was feasible and could be a simple and effective tool to increase users’ daily MVPA. Based on the results of this study, we are motivated to conduct a confirmatory study with a broader and larger number of participants.

## Abbreviations

MPA: moderate physical activity
MVPA: moderate-to-vigorous physical activity

## Data Availability

The data sets generated during or analyzed during this study are available from the corresponding author upon reasonable request.
